# The XEC Variant: Genomic Evolution, Immune Evasion, and Public Health Implications

**DOI:** 10.3390/v17070985

**Published:** 2025-07-15

**Authors:** Alaa A. A. Aljabali, Kenneth Lundstrom, Altijana Hromić-Jahjefendić, Nawal Abd El-Baky, Debaleena Nawn, Sk. Sarif Hassan, Alberto Rubio-Casillas, Elrashdy M. Redwan, Vladimir N. Uversky

**Affiliations:** 1 Department of Pharmaceutics and Pharmaceutical Technology, Faculty of Pharmacy, Yarmouk University, Irbid 21163, Jordan; 2 PanTherapeutics, Rte de Lavaux 49, CH1095 Lutry, Switzerland; 3 Department of Genetics and Bioengineering, Faculty of Engineering and Natural Sciences, International University of Sarajevo, Hrasnicka Cesta 15, 71000 Sarajevo, Bosnia and Herzegovina; ahromic@ius.edu.ba; 4 Protein Research Department, Genetic Engineering and Biotechnology Research Institute (GEBRI), City of Scientific Research and Technological Applications (SRTA-City), New Borg El-Arab City, Alexandria P.O. Box 21934, Egypt; nawalabdelbaky83@gmail.com (N.A.E.-B.); lradwan@kau.edu.sa (E.M.R.); 5 Indian Research Institute for Integrated Medicine (IRIIM), Unsani, Howrah 711302, West Bengal, India; debaleena.nawn@gmail.com; 6 Department of Mathematics, Pingla Thana Mahavidyalaya, Maligram, Paschim Medinipur 721140, West Bengal, India; sksarifhassan@pinglacollege.ac.in; 7 Autlan Regional Hospital, Jalisco Health Services, Autlan 48900, JAL, Mexico; alberto110966@gmail.com; 8 Biology Laboratory, Autlan Regional Preparatory School, University of Guadalajara, Autlan 48900, JAL, Mexico; 9 Department of Biological Sciences, Faculty of Science, King Abdulaziz University, Jeddah 21589, Saudi Arabia; 10 Center of Excellence in Bionanoscience Research, King Abdulaziz University, Jeddah 21589, Saudi Arabia; 11 Department of Molecular Medicine, Morsani College of Medicine, University of South Florida, Tampa, FL 33612, USA; vuversky@usf.edu

**Keywords:** XEC variant, SARS-CoV-2, Omicron sublineage, immune evasion, vaccine efficacy, pandemic preparedness

## Abstract

Narrative review synthesizes the most current literature on the SARS-CoV-2 XEC variant, focusing on its genomic evolution, immune evasion characteristics, epidemiological dynamics, and public health implications. To achieve this, we conducted a structured search of the literature of peer-reviewed articles, preprints, and official surveillance data from 2023 to early 2025, prioritizing virological, clinical, and immunological reports related to XEC and its parent lineages. Defined by the distinctive spike protein mutations, T22N and Q493E, XEC exhibits modest reductions in neutralization in vitro, although current evidence suggests that mRNA booster vaccines, including those targeting JN.1 and KP.2, retain cross-protective efficacy against symptomatic and severe disease. The XEC strain of SARS-CoV-2 has drawn particular attention due to its increasing prevalence in multiple regions and its potential to displace other Omicron subvariants, although direct evidence of enhanced replicative fitness is currently lacking. Preliminary analyses also indicated that glycosylation changes at the N-terminal domain enhance infectivity and immunological evasion, which is expected to underpin the increasing prevalence of XEC. The XEC variant, while still emerging, is marked by a unique recombination pattern and a set of spike protein mutations (T22N and Q493E) that collectively demonstrate increased immune evasion potential and epidemiological expansion across Europe and North America. Current evidence does not conclusively associate XEC with greater disease severity, although additional research is required to determine its clinical relevance. Key knowledge gaps include the precise role of recombination events in XEC evolution and the duration of cross-protective T-cell responses. New research priorities include genomic surveillance in undersampled regions, updated vaccine formulations against novel spike epitopes, and long-term longitudinal studies to monitor post-acute sequelae. These efforts can be augmented by computational modeling and the One Health approach, which combines human and veterinary sciences. Recent computational findings (GISAID, 2024) point to the potential of XEC for further mutations in under-surveilled reservoirs, enhancing containment challenges and risks. Addressing the potential risks associated with the XEC variant is expected to benefit from interdisciplinary coordination, particularly in regions where genomic surveillance indicates a measurable increase in prevalence.

## 1. Introduction

The onset of the COVID-19 pandemic in 2020 was a wake-up call for the scientific community and global society, resulting in more than 777 million infected individuals and 7 million deaths worldwide [1,2,3]. Severe acute respiratory syndrome coronavirus 2 (SARS-CoV-2) was rapidly isolated, and its genome was sequenced and identified as the cause of COVID-19 [4]. Various hypotheses have been proposed regarding the origin of SARS-CoV-2, including zoonotic spillover from infected wildlife at the Huanan seafood market in Wuhan and, more controversially, a potential accidental release from a laboratory. However, the most scientifically supported explanation remains zoonotic transmission, as recent high-resolution genomic tracing studies have demonstrated the co-occurrence of SARS-CoV-2-positive environmental samples with genetic material from susceptible market animals, strongly supporting natural spillover as the origin of the pandemic [5,6,7].

Before the emergence of SARS-CoV-2, epidemics occurred in various parts of the world in 2002–2003 for SARS-CoV [8]. The rapid spread of SARS-CoV-2 on all continents required drastic action, leading to severe lockdowns worldwide [9]. There has also been enormous demand for the development of novel drugs and vaccines against COVID-19. The unprecedented development of vaccines has drastically changed the course of COVID-19, leading to the emergency use authorization (EUA) of vaccines based on mRNA [10,11], DNA [12], proteins or peptides [13], viral vectors [14,15], and whole viruses [16]. After two years of mass vaccinations, the pandemic was downgraded to an endemic status [16], allowing life to return to a normal pre-pandemic status.

Since its emergence, SARS-CoV-2 has continuously evolved, giving rise to numerous lineages and sublineages that differ in terms of transmissibility, immune evasion, and geographic spread. These variants are classified by the World Health Organization (WHO) as Variants of Concern (VoC), Variants of Interest (VoI), and Variants Under Monitoring (VuM), depending on their epidemiological and virological attributes [17]. The classification system is further supported by lineage assignment frameworks, such as Pango, which enable the real-time tracking of variant evolution using genomic sequence data [18]. The mechanisms underlying the emergence of new variants driven by mutations in the spike (S) protein and immune selection pressure have been well characterized in recent reviews [19]. Against this backdrop, the XEC variant has emerged as a recombinant sublineage of Omicron that warrants focused investigation because of its potential for immune escape and epidemiological expansion. This review aims to provide a comprehensive synthesis of the current knowledge on XEC’s genetic, clinical, immunological, and public health characteristics [20].

Unsurprisingly, novel mutant SARS-CoV-2 variants emerged within a year of the onset of the pandemic. This was foreseen, as SARS-CoV-2, like other RNA viruses, is prone to mutate [21], although the mutation frequency for SARS-CoV-2 is modest compared to many other RNA viruses [22]. Typically, the variants can affect infectivity and pathogenicity, which will certainly have an impact on vaccine efficacy, as demonstrated in numerous studies [21,22,23]. Therefore, several approved vaccines have been re-engineered to better suit the modified SARS-CoV-2 variants. In this context, re-engineered vaccines have been designed to target variants such as Alpha, Beta, Gamma, Delta, and, particularly, the more recent Omicron variant [24]. Recently, the Omicron XEC subvariant [24] has emerged, which is the focus of this review. Despite being classified as a VuM, XEC warrants focused attention because of the convergence of recombination events, immune-relevant spike mutations, and the rising prevalence in over 29 countries. These attributes, combined with its escape profile in pseudovirus and live virus studies, suggest functional differences from co-circulating subvariants, particularly KP.2 and KP.3, thereby supporting the scientific rationale for this review.

Despite the emergence of numerous SARS-CoV-2 variants, the virus exhibits a relatively slow evolutionary rate compared with other RNA viruses, such as HIV-1 and influenza. This is primarily due to the proofreading activity of the viral RNA-dependent RNA polymerase complex, which reduces the mutation frequency [25]. As a result, the majority of mutations observed in VoC are non-synonymous and often exert minimal effects on viral replication fitness [26]. While specific mutations, particularly in the spike protein, can enhance transmissibility or immune evasion, these changes remain limited in scope and typically occur against a backdrop of overall genomic stability. This constrained evolutionary landscape is expected to partly explain the limited divergence among Omicron sublineages, including the XEC.

### Review Methodology

This narrative review was conducted to cover the current evidence related to the SARS-CoV-2 XEC variant. A comprehensive search of the literature was performed using PubMed, Scopus, Web of Science, bioRxiv, medRxiv, Preprints, GISAID, and official surveillance sources from the WHO and CDC. Searches were conducted using combinations of keywords such as “XEC variant,” “SARS-CoV-2 sublineage,” “immune evasion,” “genomic recombination,” and “variant under monitoring.” Sources were included if they provided primary data, surveillance reports, or mechanistic insights related to virology, evolution, epidemiology, clinical impact, or immune response associated with XEC and its parent lineages (KS.1.1, KP.3.3, KP.3.1.1). Preprints were included only if supported by cited peer-reviewed studies or if widely referenced in WHO or CDC reports. Studies were appraised for scientific credibility, methodological transparency, and relevance. Contradictory findings were presented side by side to maintain scientific neutrality and avoid narrative bias.

To ensure the robustness of our review process, we applied rigorous inclusion criteria, prioritizing peer-reviewed primary studies, reports with experimental validation, and high-confidence genomic surveillance data. Conflicting findings were analyzed in parallel, and interpretations were limited to evidence-supported patterns to avoid biases. Wherever conclusions are drawn in this review, they are explicitly limited to the scope of currently available genomic, immunological, or surveillance data; speculative interpretations have been avoided or clearly labeled as such. To preserve objectivity, all interpretations in this review were directly anchored to published peer-reviewed studies or official surveillance reports. Speculative content has been clearly identified and qualified.

## 2. Genomic Landscape

The XEC variant was first identified in Germany in mid-2024 through genomic surveillance of recombinant Omicron sublineages [27,28]. It originated from the recombination of the KS.1.1 and KP3.3 variants, which are descendants of the Omicron variant and closely related to the globally dominant JN.1 variant [29]. The XEC variant has been characterized by its enhanced relative effective reproduction [30], making it a potential candidate for outcompeting other SARS-CoV-2 lineages [31]. The XEC variant carries a relatively rare T22N mutation in the SARS-CoV-2 S protein from KS.1.1 and a Q493E mutation from KP3.3 [31]. Although the T22N mutation has not been thoroughly investigated, the Q493E mutation has been associated with an enhanced binding affinity for the ACE2 receptor, rendering XEC potentially more infectious. The combination of the Q493E mutation with other mutations in the receptor-binding domain (RBD) can potentially contribute to the enhanced evasion of immune responses [31].

Phylogenetic analyses of XEC genomes submitted to GISAID indicate a close relationship with the KP.3.3 parental lineage, which harbors the highest number of characteristic mutations [17,32,33]. Furthermore, the application of the FUBAR algorithm for selection pressure tests indicated that the sites under selection were predominantly located in the S protein, providing a significant portion of the genetic variability [34]. Therefore, it is anticipated that new mutations will modify the genetic makeup of the XEC variant, resulting in new variants replacing the most common JN.1 variant [3]. For the above-mentioned reasons, the phylogeny of the parental lineages and the XEC strain can be considered an evolutionary dead-end phenomenon, which most likely does not have the capacity to result in a wider global presence. Although XEC is expected to show superior infectivity, there is no indication that it causes enhanced disease symptoms or lethality. Moreover, elevated genomic diversity is not indicative of increased severity or danger of the virus. Even if new variants emerge, T-cells should continue to provide significant protection. Despite this, genome-based surveillance is necessary to monitor the evolutionary processes of viruses and correctly respond to any potential threats. According to another study, XEC has the potential to outcompete other major SARS-CoV-2 lineages [34]. For instance, XEC has shown higher pseudovirus infectivity and immune evasion than KP.3. [34]. To further delineate the functional relevance of XEC, Table 1 summarizes its defining spike protein mutations, source lineages, and experimentally supported or computationally modeled roles in viral behavior.

It is important to note that the classification of SARS-CoV-2 variants, particularly VuM, often reflects a combination of short-term epidemiological shifts and precautionary public health measures. In the case of XEC, although it demonstrates increased relative infectivity and immune escape characteristics, the degree of genomic divergence remains limited. This suggests that the variant’s classification is influenced as much by its transient epidemiological prominence and recombination patterns as by its biological novelty. Therefore, careful evaluation of both functional impacts and evolutionary trajectories is essential when assigning variant status, especially given the generally slow evolutionary rate of SARS-CoV-2 compared to other RNA viruses.

## 3. Epidemiological Dynamics

Transmission and Prevalence: XEC has demonstrated a notable growth advantage over previous subvariants, leading to its rapid spread across multiple regions worldwide. By late 2024, XEC accounted for approximately 4.8% of the SARS-CoV-2 sequences submitted to GISAID globally, with Germany and the United States among the countries reporting the highest number of sequences [1,37]. According to WHO and CDC reports from late 2024, XEC increased from 2.0% to 36.8% of global GISAID sequences over 13 weeks, outpacing KP.2 and approaching the trajectory previously observed with KP.3.1.1. This sustained increase in prevalence reflects more than just a transient spread. This heightened transmissibility is attributed to genetic recombination, which is expected to enhance its ability to evade immune responses.

Clinical Manifestations: The clinical presentation of XEC infections is consistent with that of previous Omicron subvariants. The common symptoms include fever, cough, nasal congestion, headache, and fatigue. Notably, there is currently no evidence to suggest that XEC causes more severe illness than its predecessors.

Public Health Implications: The emergence of XEC underscores the importance of continuous genomic surveillance to monitor SARS-CoV-2 evolution. Public health authorities should maintain robust surveillance systems for influenza-like illnesses and severe acute respiratory syndrome to promptly detect and respond to such variants. Although the XEC subvariant exhibits increased transmissibility, the current data do not indicate a heightened disease severity. While definitive conclusions on long-term dominance remain premature, the unique combination of immune escape markers, recombination origin, and rapid regional expansion supports the WHO’s decision to classify XEC as a VuM. These attributes signal a potential shift in the landscape of immune adaptation and justify a targeted investigation from both virological and public health perspectives. Ongoing monitoring and vaccination efforts are crucial for managing its spread and impact on public health. The key SARS-CoV-2 variants circulating worldwide are JN.1, BA.2.86, KP.3, KP.2, and XEC, all of which are Omicron subvariants [1]. In September 2024, the WHO designated it as a VuM [17]. From the time XEC was detected until epidemiological week 37 of 2024, it spread to over 27 nations across North America, Europe, and Asia. As of mid-September, the highest number of XEC cases had been identified in Germany, the United States, France, Denmark, and the UK.

Currently, the WHO is monitoring several SARS-CoV-2 VuM, including JN.1.18, KP.2, KP.3, KP.3.1.1, LB.1, and XEC. Among these, XEC has shown the most notable increase in prevalence. Both XEC and KP.3.1.1 demonstrated continuous increases in global prevalence from Epiweek 34 through Epiweek 47 of 2024, although at different growth rates, whereas other VuM declined steadily over the same period (Table 2). Specifically, XEC rose from 2.0% in Epiweek34 to 36.8% in Epiweek 47, whereas KP.3.1.1 increased from 34.6% to a peak of 48.8% in Epiweek 40 before gradually declining to 41.8% by Epiweek 47 (Table 2).

**Figure 1:**
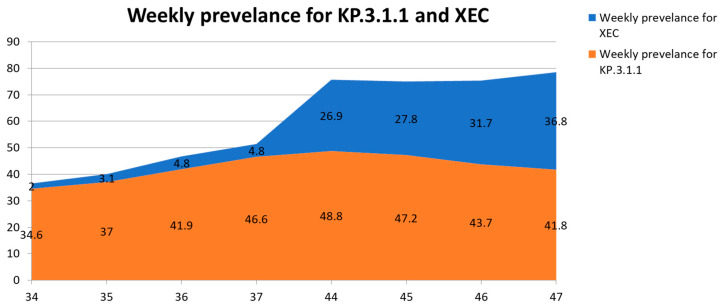
Weekly global prevalence of SARS-CoV-2 variants XEC and KP.3.1.1 from Epiweek 34 to Epiweek 47 of 2024. Stacked area chart displaying the relative weekly proportions of GISAID-submitted sequences identified as XEC (blue) and KP.3.1.1 (red) at eight surveillance time points. Data were derived from the WHO variant tracking summaries using GISAID aggregate reports between 19 August and 7 December 2024. XEC prevalence rose steadily from 2.0% in Epiweek 34 to 36.8% by Epiweek 47, while KP.3.1.1 initially increased to 48.8% in Epiweek 44 before gradually declining to 41.8%. Data was generated from [17,33,38,39].

In Epiweek 47 (18–24 November 2024), 50 countries submitted 13,331 XEC sequences to GISAID [17], accounting for 36.8% of the global sequences. These data suggest a noteworthy increase in the prevalence from 4.8% in Epiweek 37 to 26.9% in Epiweek 44 (28 October to 3 November 2024), as illustrated in Table 2 and Figure 1. In contrast, KP.3.1.1 declined in prevalence from 48.8% in Epiweek 44 to 41.8% in Epiweek 47 (Table 2 and Figure 1), respectively. On 1 November 2024, the National Center for Immunization and Respiratory Diseases of the CDC announced that XEC would increase as KP.3.1.1 decreased [40]. XEC and KP.3.1.1 epidemiological dynamics also displayed remarkable regional variances [3]. Between Epiweeks 34 and 37 of 2024, the prevalence of XEC increased steadily in Europe (from 5.3% to 12.0%) and the Americas (from 0.9% to 2.8%), with modest gains in the Western Pacific region (from 0.2% to 2.0%). In contrast, KP.3.1.1 demonstrated more pronounced growth across the same period in the Western Pacific (13.5% to 24.2%) and the Americas (34.1% to 49.2%), while remaining virtually undetected in Southeast Asia, where only a single sequence was reported (Table 3).

The noteworthy increase in the prevalence of XEC between Epiweeks 44 and 47, along with the steady decrease in KP.3.1.1 prevalence (formerly the most prevalent variant) globally, suggests that XEC is expected to become a leading subvariant, depending on future transmission patterns of SARS-CoV-2. However, XEC still has a minimal antigenic advantage in escaping preceding immunity, which maintains a low overall risk evaluation for this Omicron subvariant (Table 4) [3]. As of Epiweek 47 of 2024, the XEC variant of SARS-CoV-2 had been identified in at least 28 countries, with the highest absolute counts observed in the United States (n = 168), Canada (n = 140), and the UK (n = 122). Several European countries, including France, Germany, and Sweden, reported notable sequence numbers, whereas detections in East Asia and South America remained limited to isolated cases (Table 3). Furthermore, as illustrated in Figure 1, XEC demonstrated a marked rise in global prevalence from 2.0% in Epiweek 34 to 36.8% by Epiweek 47, overtaking several co-circulating variants. KP.3.1.1, in contrast, peaked at 48.8% in Epiweek 44 before exhibiting a moderate decline in the subsequent weeks.

### 3.1. Global Distribution Patterns

After being identified in August 2024 in Berlin, Germany, in COVID-19 samples, XEC emerged in the UK on 18 September. On 26 October, XEC was spreading in the UK at a high rate (approximately 7% of cases), as stated by the UK Health Security Agency (UKHSA) [41]. On 20 September 2024, GISAID data revealed that more than 600 XEC cases had been identified across 27 countries and that XEC was most prevalent in Europe, identified in no less than 13 nations [28]. This subvariant was the most widespread in France, involving approximately 21% of the sequenced cases of COVID-19. In contrast, approximately 1% of XEC cases were detected in the United States in a week [42].

The heightened transmissibility is attributed to genetic recombination, which is expected to enhance its ability to evade immune responses. WHO surveillance data from late 2024 showed that XEC was designated a VuM in September 2024 and had spread to over 27 countries by epidemiological week 37, including Germany, France, the United States, Denmark, and the UK [2,28]. Despite its confirmed spread to over 27 countries and a notable genomic presence in Germany by October 2024, XEC has received limited public attention. For instance, it was not mentioned in the Robert Koch Institute’s COVID-19 assessment published on 18 September 2024. In Germany, attention is still paid to the most dominant variant, KP.3.1.1, which is considered more transmissible than previous variants. However, by October 2024, XEC had its highest concentration in Germany, with a genomic prevalence of approximately 13% [43].

By mid-October 2024, XEC had been detected in no less than 29 countries across the globe and 24 states in the United States [44]. This rapid spread between nations around the world is indicative of the highly contagious potential of this Omicron subvariant (Table 5). On 10 October 2024, it was stated that XEC also hit Australia [45]. The Australian Respiratory Surveillance Report announced that there had been an increased percentage of recently sequenced XEC cases. Approximately 329 sequences of SARS-CoV-2 were collected from 26 August to 22 September and uploaded on the national genomics surveillance platform of Australia (AusTrakka) for COVID-19. It turned out that 91.5% of these were KP.2 and KP.3, and 8.5% were recombinant sublineages of Omicron, including XEC [46]. The African CDC published a statement on the XEC subvariant on 4 November 2024, revealing that one XEC case was reported in Botswana from a hospitalized traveler from Europe [47]. The agency also declared that limited sequencing and testing, compared to previous levels, made it challenging to identify the XEC spread in Africa [48]. It was stated that XEC is a recombinant subvariant under monitoring by the African CDC in addition to the WHO [49]. As shown in Table 4, both XEC and KP.3.1.1 exhibited relatively uniform distribution across age groups, with the highest proportions observed in adults aged 60–79 (32.4% and 33.1%, respectively). By contrast, other Omicron variants were more frequently detected in children aged 0–19, while recombinant lineages were rare across all age categories.

On 23 December 2024, the Ontario COVID-19 Genomics Network revealed in its weekly epidemiological summary on whole genome sequencing of SARS-CoV-2 that 811 cases were sequenced between 1 December and 7 December 2024, 34.6% of which were XEC and 25.2% were KP.3.1.1 [50]. The report also demonstrated that XEC remained steady at 34.9% (24–30 November) and 34.6% (1–7 December), whereas KP.3.1.1 declined from 30.8% (24–30 November) to 25.2% (1–7 December) [17,32,33,38,39].

### 3.2. Transmission Efficiency

Although XEC is still a minority variant of SARS-CoV-2 worldwide, it seems to exhibit more spread than other circulating variants [3]. It spread persistently across the world in 2024. Hence, it is suggested that XEC can outpace the other variants in the coming months. Unquestionably, XEC seems to have an advantage compared to other variants at present, considering its increased percentage of cases. Nonetheless, XEC does not resemble Omicron, which significantly altered the trajectory of the pandemic in ways that were not fully understood at the time. XEC surpasses the other two Omicron subvariants, KP2 and KP3, which were already more transmissible or superior at escaping the immune system than Omicron due to the slight differences in their S proteins [40].

Initial investigational records proposed that XEC displayed distinctive mutations and improved transmissibility, which is expected to cause a comparatively greater evasion of the immune system than KP.3 (parent lineage) [51,52,53]. Nevertheless, there has been no confirmation of a more severe infection in XEC cases than seen in preceding variants of Omicron. The CDC in the United States noted that XEC can escape immunity, and it is more immune-evasive than previous Omicron sublineages [43].

Antigenic drift or mutations result in new SARS-CoV-2 variants that appear to alter the immune system. As XEC is primarily based on the recombination between two JN.1 descendant variants, it can escape immunity and cause disease. What makes this subvariant of Omicron more transmissible are the Q493E and T22N mutations in its S protein [31]. The investigation of pseudoviruses has revealed the XEC-enhanced evasion of humoral immunity, which arises from the conformational dynamics in the RBD prompted by the T22N mutation [31,36]. Additionally, studies on live viruses confirmed a substantial antibody titer decline from XBB.1.5 and B.1 to XEC and KP.3.1.1 in 68–82-year-old individuals from Norway [54].

### 3.3. Comparative Transmissibility Metrics

Regarding growth advantage, the WHO considers XEC a high-level risk, since this subvariant is spreading considerably across all regions with the steady sharing of sequence data of SARS-CoV-2, while KP.3.1.1 (the previously most prevalent variant) is beginning to decline [17]. Between Epiweeks 44 and 47, XEC demonstrated an increase from 37.0% to 48.0% in Europe, 14.3% to 35.6% in the Western Pacific region, and from 22.7% to 32.8% in the Americas. On the other hand, only four XEC sequences were detected in the Eastern Mediterranean and African regions, and seventeen sequences in the Southeast Asia region [17]. In August 2024, XEC’s Re of XEC was 1.13-fold greater than that of KP.3.1.1 [35]. As of 3 September, KP.3.1.1 was detected 14,396 times worldwide, KP.3.3 9 157 times, K.S.1.1 2650 times, and XEC 95 times (according to data provided by GISAID) [55]. This reflects the dominance of KP.3.1.1. Table 6 shows that XEC and KP.3.1.1 were similarly distributed across age groups, with detection rates above 30% in most categories, particularly among adults aged 60–79 and 80 years and older. In contrast, other Omicron lineages were more commonly identified in younger individuals aged 0–19, while recombinant variants remained rare in all age brackets, accounting for around 1% or less overall.

## 4. Clinical and Immunological Insights

### 4.1. Symptom Profile Variations

XEC, a recombinant of the Omicron subvariants KS.1.1 and KP3.3, has demonstrated a spread advantage over prior strains, raising concerns about its potential to surpass dominant variants. Notably, its T22N and F59S mutations in the N-terminal domain (NTD) of the S protein are expected to enhance immune evasion, with T22N creating a possible N-linked glycosylation site similar to the DelS31 mutation in KP.3.1.1 [56]. These evolutionary changes are expected to impact transmissibility and severity [29].

Available clinical data on XEC infections is limited. Preliminary reports suggest that symptoms are generally mild to moderate, like those caused by other Omicron subvariants. Common manifestations include fever, cough, fatigue, and sore throat, although cases of shortness of breath and prolonged symptoms have also been reported in vulnerable individuals. No definitive evidence exists to suggest that XEC causes more severe disease than its parental lineage [39]. This is consistent with prior research revealing differences in symptom intensity and duration across SARS-CoV-2 variants [57]. Although the symptom profile is similar, some experts believe that the XEC variant is expected to have more severe flu-like symptoms than its predecessors [58]. According to reports, patients are expected to experience an increased intensity of symptoms, such as bodily pain and weariness, which might lead to exhaustion [59,60]. However, there is no compelling evidence that XEC produces more severe illness than the Alpha or Delta variants, which have been associated with greater hospitalization rates and lethal outcomes. Patients recovering from Omicron infections have also reported long-term COVID-19 symptoms, such as chronic tiredness and cognitive impairment [61].

### 4.2. Immune Response Characteristics

Li et al. [29] determined that XEC shows a higher infectivity than the parental KP.3 variant, although it is still lower than that of the KP.3.1.1 variant in both HEK293T-ACE2 and Calu-3 cells [62]. This conclusion is consistent with the findings from other studies, which found that the XEC variant showed reduced infectivity compared to the KP.3.1.1 variant in HOS-ACE2-TMPRSS2 [30] and Calu-3 cells [63], as well as comparable infectivity in Vero cells. They also discovered that the single mutation F59S in the NTD accounts for most of the enhanced infectivity, but the T22N mutation in the same domain has no substantial influence, which is supported by other studies [30,62]. Deep mutational scanning of the XBB.1.5 S protein indicated that the F59S mutation might provide a moderate increase in ACE2 binding [64]. However, other studies have shown comparable ACE2 binding across the KP.3, KP.3.1.1, and XEC variants [63,65].

Mutations in the NTD that produce additional glycosylation sites are common for both XEC and KP.3.1.1 [66]. The DelS31 mutation in KP.3.1.1 is projected to result in a glycosylation site at residue N30, whereas the T22N mutation in XEC is likely to result in a glycosylation site at residue N22. According to Li et al. [29], ablating the glycosylation site in KP.3.1.1 significantly reduced infectivity while partially restoring neutralization sensitivity, particularly in bivalent vaccine recipients within BA.2.86/JN.1 patient cohorts [63]. These findings are supported by Liu et al., who observed that glycosylation in XEC can be inhibited by soluble ACE2 and RBD-targeting monoclonal antibodies [65]. These results suggest that NTD glycosylation plays a significant role in spike stability, viral infectivity, and neutralizing antibody (nAb) activities. The NTD has no direct contact with the ACE2 receptor, but it is critical for maintaining the shape and dynamics of the protein. Homology modeling revealed that T22N and F59S mutations in the NTD of the XEC S protein are expected to influence spike stability and viral infectivity; however, the precise mechanisms require additional experimental confirmation. The T22N mutation creates an N-linked glycosylation site at position 22, which is expected to impair antibody identification and promote immune evasion [29].

According to recent research, the XEC variant possesses mutations in its S protein, including F59S and Q493E, which are critical for infectivity. The Q493E mutation is particularly significant because it is associated with a higher binding affinity for the ACE2 receptor, allowing for more viral entry into host cells [53]. This mutation, along with others acquired from its parental lineages, enables XEC to avoid neutralizing antibodies produced by previous infections or immunizations [56]. Studies have shown that XEC is much less neutralized by antibodies generated from patients previously infected with KP.3.1.1 but is more resistant to sera from those individuals infected with KP.3.3 [53]. This suggests a strong immune evasion capability since mutations in the RBD can synergistically improve both the binding affinity and immunological escape [53]. Furthermore, the extent of genetic diversity in the S protein implies that lasting evolution is expected to continue to favor such mutations, potentially increasing the prevalence of the XEC subvariant and its presence over other variants [67].

### 4.3. Potential Impacts on Vaccine Effectiveness

Arora et al. [63] described the virological characteristics of the XEC lineage and investigated the effect of the JN.1 booster vaccination on KP.3.1.1 and XEC neutralization. They discovered that the XEC S protein engaged ACE2 with the same efficacy as the JN.1 and KP.3.1.1 S proteins. The cell entry was analyzed using S protein-bearing pseudovirus particles, which is a well-established surrogate method for studying SARS-CoV-2 cell entry and neutralization. Pseudovirus particles containing JN.1 (JN.1pp), KP.3.1.1 (KP.3.1.1pp), or XEC S proteins (XECpp) entered Vero cells with equal efficiency, whereas KP.3.1.1pp and XECpp had a lower entry rate into Calu-3 lung cells [63]. In the same study, the JN.1-adapted mRNA vaccine, bretovameran (developed by Pfizer-BioNTech), enhanced neutralization against the SARS-CoV-2 variants KP.3.1.1 and XEC. In a group of 33 vaccinated individuals, neutralization increased dramatically after the booster, with geometric mean titers (GMT) of 2430 for JN.1, 1300 for KP.3.1.1, and 840 for XEC, respectively. Nevertheless, the neutralization of KP.3.1.1 and XEC was lower than that of JN.1. In a second cohort of newly infected people without the booster, JN.1 neutralization was similarly more successful than against other variants, demonstrating that vaccination effectiveness against emerging variants is expected to be challenging [63]. Overall, XEC has shown stronger pseudovirus infectivity and immune evasion than KP.3. XEC demonstrated comparatively stronger immunological resistance than KP.3.1.1 in early pseudovirus assays, implying that the greater Re of XEC than KP.3.1.1 is due to this feature and that XEC will soon be the most common SARS-CoV-2 variant worldwide.

Notably, the T22N mutation in the NTD and the Q493E mutation in the RBD of the XEC S protein are associated with immune evasion. In general, mutations within the RBD can alter the structural conformation required for neutralizing antibody recognition, thereby reducing the effectiveness of the immune response. As a result, vaccinated individuals are expected to show a reduced protection against infection, although they are expected to still show some immunity against severe COVID-19 [68].

The ability of XEC to partially escape immune responses raises concerns about the possible occurrence of breakthrough infections. As vaccination coverage expands globally, the possibility of encountering variants, such as XEC, is expected to increase the rates of reinfection among vaccinated persons. This scenario has already been described with other variants, in which increased transmissibility mixed with immunological escape has resulted in the increase in prevalence, despite high vaccination rates. Given the decreased neutralizing efficacy of antibodies toward XEC, there is an urgent need for ongoing research on next-generation vaccines that can elicit broader immune responses effective against novel variants.

## 5. Future Aspects

As the XEC variant continues to circulate at low to moderate levels globally, future studies are needed to assess its immune escape potential in vaccinated populations, as well as to clarify the durability of cross-protection conferred by mRNA booster vaccines. Improved recombinant lineage monitoring and region-specific immunosurveillance will be key to determining the long-term relevance of XEC within the SARS-CoV-2 evolutionary landscape.

### 5.1. Critical Knowledge Gaps

#### 5.1.1. Variant-Specific Virological Characteristics

The mutational pattern of the XEC variant raises questions regarding its biological behavior. Notably, the co-occurrence of T22N and Q493E within functionally constrained regions supports a plausible model for synergistic immune evasion, which is now observed across multiple recombinant lineages with epidemiological significance. S protein mutations, especially in the RBD, the furin cleavage site, and the NTD, are thought to increase the ACE2 binding affinity, alter cell tropism, and potentially facilitate syncytia formation [29,46]. Mutations at glycosylation sites in the NTD can potentially affect immune evasion and infectivity, although the exact mechanisms are poorly understood [31,69].

Structural characterization through cryo-EM or X-ray crystallography remains limited, and the major antigenic epitopes of XEC are poorly characterized. It also remains unknown whether XEC arose through recombination events between circulating lineages or prolonged intra-host evolution, and it is necessary to clarify its evolutionary history for the predictive modeling of emerging variants [70].

#### 5.1.2. Immune Evasion Dynamics

A central concern with XEC is its potential to evade preexisting immunity. While early data indicate reduced neutralization by previously induced infection or vaccine antibodies, the extent of escape from hybrid immunity (post-vaccination and infection) and next-generation vaccines for Omicron subvariants, such as XBB.1.5, needs to be elucidated [71,72]. Furthermore, T-cell responses are expected to be compromised if XEC carries mutations that alter the conserved epitopes. Recent studies have also indicated that NTD glycosylation alterations can confer additional immune escape advantages [29]. However, detailed examinations of the protective thresholds for neutralizing antibodies, mucosal IgA responses, and T-cell cross-protection remain incomplete.

#### 5.1.3. Pathogenesis and Clinical Impact

The pathogenicity of XEC compared to earlier variants is a continuing line of research. There are open questions regarding whether XEC shows increased replication in lower respiratory tissue, which would aggravate disease severity, or whether it expresses neurotropic tendencies that would be responsible for the neurological complications observed in some cases of COVID-19. In addition, its association with the post-acute sequelae of SARS-CoV-2 infection (PASC) or long COVID has yet to be explored [73]. There is evidence of a shift in the clinical presentation and severity of disease caused by variants of the Omicron lineage [74]. The extent to which these changes apply to XEC requires new clinical indicators and biomarkers. The established clinical indicators are expected to be invalid, indicating the need for new diagnostic and prognostic tools.

#### 5.1.4. Transmission and Epidemiologic Fitness

XEC appears to have a transmissibility advantage in some places; however, the mechanisms of its rapid spread are not fully understood. These geographical regions include aerosol stabilization, heightened aerosol transmissivity during asymptomatic or presymptomatic carriage, and super-spreader events facilitated by specific social behaviors. The accurate measurement of its reproduction number (Rt) and the identification of transmission hotspots for XEC are crucial for the design of evidence-based containment strategies [33,69].

#### 5.1.5. Geographic and Host-Specific Heterogeneity

Discrepancies in surveillance hinder the construction of a complete picture of the global distribution of XEC. Low-resource areas, immunocompromised patients, and suspected animal reservoirs including wildlife and domesticated animals continue to be insufficiently sampled [75]. The process of reverse spillovers to wildlife can lead to secondary reservoirs, making eradication even more challenging. The different patterns of immunity across various human populations underscore the need for analysis in each context to facilitate fair healthcare planning [76].

#### 5.1.6. Long-Term Sequelae and Psychosocial Impact

The long-term effects of XEC infection, especially in patients with underlying health conditions, should be carefully monitored. Recent SARS-CoV-2 variants have been linked to long-term COVID-19 and post-acute sequelae, sparking fears that XEC produces similar or even more serious aftereffects [73]. In addition, the emergence of new variants often brings heightened public concerns, making it even more crucial to address the psychosocial aspects of XEC using evidence-informed communication strategies and mental health support programs [77].

### 5.2. Emerging Research Priorities

#### 5.2.1. Enhanced Molecular Surveillance

The worldwide expansion of genomic surveillance networks, most critically in low-resource settings, is crucial for tracking the dissemination of XEC and detecting new SARS-CoV-2 variants and subvariants [78]. Wastewater surveillance is an affordable supplement to clinical diagnosis, enabling the early detection of community outbreaks. The combination of AI-based predictive modeling with up-to-date sequencing data can greatly enhance situational awareness and allow for timely public health action [42,79,80,81].

#### 5.2.2. In Vitro and In Vivo Models

There is a need to define sufficient experimental models to study the infectivity and pathogenicity of XEC. Pseudovirus-neutralizing assays using the sera of vaccine recipients, convalescents, and booster recipients can yield estimates of protection efficacy provided by antibodies. Human airway organoids, in conjunction with other models, such as hamsters and ACE2-transgenic mice, are crucial for investigating replication kinetics, tropism to different tissues, and concomitant clinical presentations [82,83].

#### 5.2.3. Immune Correlates of Protection

There is a need to define neutralizing antibody protection thresholds and mucosal IgA immunity to assess vaccine efficacy. In parallel, the identification of conserved CD8+ T-cell-defined epitopes in response to S protein mutations will help to define the role of cellular immunity in averting severe disease. Such observations will sharpen the immunological correlates of protection and inform the design of next-generation vaccines [84,85].

#### 5.2.4. Antiviral and Therapeutic Resistance

Assessment of the efficacy of current antiviral drugs against XEC requires careful analysis. Mutations in the primary protease (3CLpro) or RNA-dependent RNA polymerase can result in a resistance to drugs such as nirmatrelvir/ritonavir (paxlovid) or remdesivir [86]. Broad-spectrum protease inhibitors and cellular or conserved process-based therapies hold promise; however, careful validation via phase I to IV clinical studies is required [87].

#### 5.2.5. Longitudinal Clinical Studies

Population-based cohort studies are paramount in defining the clinical course of XEC in various groups of patients, including children and patients with comorbidities (e.g., diabetes or COPD). Monitoring patterns of recovery and chronic outcomes will define the wider health consequences, contribute to clinical management strategies, and improve the quality of patient counseling programs [88,89].

#### 5.2.6. Integrating Computational Approaches

Sophisticated computational systems and informative graphs hold great promise to deepen our understanding of the pathophysiological mechanisms of XEC, facilitate the expedited identification of drug targets, and predict evolutionary patterns. Using big data analysis, researchers can identify key intervention points for treatment and quickly adapt to constantly evolving patterns of viral behavior [90].

### 5.3. Potential Intervention Strategies

#### 5.3.1. Optimized Vaccine Development

The modification of mRNA vaccines to use XEC-specific S protein antigens or conserved pan-coronavirus epitopes is a logical next step. In addition, intranasal vaccine strategies, such as protein nanoparticles or adenoviral vectors, provide the added benefit of inducing mucosal immunity, which is the first line of defense against respiratory infections. Further use of self-replicating RNA-based vaccines will allow the administration of reduced vaccine doses, potentially decreasing adverse events and lowering vaccine production costs [75]. Proper regulation and robust manufacturing infrastructure will be crucial for enabling quick deployment [91,92].

#### 5.3.2. Next-Generation Antivirals

Broad-spectrum protease inhibitors targeting highly conserved viral enzymes such as 3CLpro, when combined with host-targeted pharmacological drugs such as TMPRSS2 inhibitors, can help curtail the resistance developed against variants [86]. The use of direct-acting antivirals in combination with drugs that affect host factors can greatly reduce resistance, especially in immunocompromised patients receiving protracted treatment regimens [93].

#### 5.3.3. Monoclonal Antibody Therapies

There is a need to engineer monoclonal antibodies (mAbs) with activity against emerging variants to reduce the severity of disease and mortality. Targeted approaches, such as yeast or phage display libraries, can identify mAbs against structurally constrained and less mutation-prone regions of the S protein. The periodic updating of therapies to match the XEC mutational profile is the basis for long-term efficacy [94,95].

#### 5.3.4. Non-Pharmaceutical Interventions (NPIs)

Physical distancing, masking, and improved indoor air quality via ventilation and filtration are effective in preventing respiratory transmission [96]. High-filtration masks (N95 and KF94) offer additional protection in high-risk settings. NPIs can be adapted to the specific features of XEC, including its transmissibility and immune escape potential, to optimize public health gains [97]. The NPIs played an important role during the COVID-19 pandemic, especially during the time when no vaccines were available. It is therefore necessary that NPIs are developed and rapidly available in case of newly emerging variants or pandemics [98].

#### 5.3.5. One Health Approach to Understanding the XEC Mutation of SARS-CoV-2

The exploration of potential reservoirs of zoonosis and reverse spillover events is key to preventing further adaptation of viruses and the resulting pandemics. Routine surveillance of wildlife, domestic animals, and ecological interfaces can help identify areas at high risk for interspecies transfer. This requires interdisciplinary cooperation between virologists, epidemiologists, veterinarians, and ecologists [99,100].

#### 5.3.6. Global Equity Initiatives and Rapid Response Systems

The XEC variant serves as yet another example of the almost never-ending adaptability of SARS-CoV-2 and presents a challenge to global public health to stem and manage the pandemic [28,43]. Filling the critical knowledge gaps from variant-specific virological properties to longer-term clinical sequelae will necessitate collective efforts across multiple disciplines, including virology, immunology, epidemiology, computational modeling, and social sciences [31,40]. Importantly, the combination of robust enhanced genomic surveillance, next-generation vaccine platforms, and integrated therapeutic strategies can all reduce the morbidity, mortality, and socioeconomic determinants of XEC [28,44].

Researchers and policymakers should not allow uncertainty to paralyze them and should instead leverage equitable healthcare measures and international collaboration to turn uncertainty into actionable insights while its advancements are deployed to the population, including to those outside their geographic or socioeconomic backgrounds [101]. If XEC continues to exhibit a distinct epidemiological or immune profile, insights from its emergence are expected to inform broader system-level improvements in pandemic preparedness. These systems will be better prepared to combat emerging pathogens and adapt to evolving health challenges [102].

## 6. Conclusions

The emergence of the XEC variant underscores the continued capability of SARS-CoV-2 to evolve and adapt, even during a global trend toward endemicity. Higher reproduction rates, adaptations of the S protein to enable immune evasion, and instances of recombination, such as the Q493E mutation, pose a challenge to vaccine development and treatment efficacy. There is currently no evidence to link XEC to increased severity or mortality, yet resistance to neutralizing antibodies combined with potential chronic reservoirs in human hosts and animals require close surveillance and quick response mechanisms.

The XEC variant represents a recombinant lineage of SARS-CoV-2 Omicron subvariants that has gained limited but measurable geographic traction. Available evidence suggests that its symptom profile closely resembles that of other Omicron sublineages, and no definitive clinical or epidemiological indicators currently support an increased severity or altered vaccine efficacy. Genomic data indicates that XEC shares several mutations with its parental lineages. While preliminary findings suggest potential functional divergence, definitive conclusions require additional validation through in vitro and epidemiological studies. Continued genomic surveillance and real-world clinical studies will be important to monitor the evolution and potential relevance of XEC, particularly in immunocompromised populations. In addition, the psychosocial impact of emerging variants in the form of heightened anxiety and changed health-seeking behavior underscores the need to enact risk communication approaches that are tailored to different demographic groups. In the future, pandemic readiness at a global scale will require a unifying effort that encompasses large-scale molecular surveillance, novel vaccine design, and more expansive studies of antiviral drugs and mAbs. The One Health approach remains a valuable framework for anticipating emerging zoonotic risks, though its direct applicability to XEC will require further investigation. By capitalizing on knowledge gained during the XEC experience and encouraging multidisciplinary cooperation between virologists, immunologists, epidemiologists, clinicians, and policymakers, the scientific community can build stronger healthcare infrastructures. In the end, such preventive efforts do not just mitigate the health threats of XEC but also provide a roadmap to a more resilient and equitable response to future public health challenges.

## Figures and Tables

**Table 1:** Key spike protein mutations in the SARS-CoV-2 XEC variant and their functional implications. This table summarizes the principal spike protein mutations observed in the XEC variant of SARS-CoV-2, detailing their structural locations, parental lineages of origin, and experimentally or computationally supported functional consequences. The T22N and F59S mutations, located in the N-terminal domain (NTD), are associated with enhanced glycosylation potential and spike stability [29], whereas the Q493E mutation in the receptor-binding domain (RBD) is linked to increased ACE2 affinity and immune escape. These mutations are expected to act synergistically to increase viral transmissibility and reduce neutralizing antibody recognition. Functional insights were based on in vitro pseudovirus infectivity assays, structural modeling, and neutralization studies, as referenced below.

Mutation	Location	Source Lineage	Functional Role	Reference
T22N	Spike (NTD)	KS.1.1	Creates glycosylation site; enhances immune evasion	[29]
F59S	Spike (NTD)	De novo	Enhances infectivity via structural changes	[35]
Q493E	Spike (RBD)	KP.3.3	Increases ACE2 affinity; immune escape	[36]

**Table 2:** Geographic spread, genomic surveillance counts, and temporal prevalence trends of WHO-designated variants under monitoring (VuMs) during Epiweeks 34–47 of 2024. The table summarizes the number of affected countries, submitted sequences (as of Epiweeks 37 and 47), and the estimated global prevalence (%) of six SARS-CoV-2 variants under monitoring (XEC, KP.3.1.1, KP.3, KP.2, LB.1, and JN.1.18) over consecutive epidemiological weeks. XEC, first flagged in September 2024, showed a marked rise in prevalence from 2.0% to 36.8% over this period, whereas KP.3.1.1 remained dominant. Data were compiled from the WHO’s weekly genomic surveillance reports. Data compiled from [38,39].

VuMs	No. of Nations	No. of Submitted Sequences		Prevalence (%)							
		Epiweek 37	Epiweek 47	Epiweek 34	Epiweek 35	Epiweek 30	Epiweek 40	Epiweek 37	Epiweek 44	Epiweek 46	Epiweek 47
XEC	29	1263	13,331	2	3.1	4.8	4.8	26.9	27.8	31.7	36.8
KP.3.1.1	54	29,557	65,234	34.6	37	41.9	46.6	48.8	47.2	43.7	41.8
KP.3	70	45,327	56,177	18.8	18.4	16.9	14.4	8.4	7.7	8.2	6.1
KP.2	82	27,976	33,287	12	10.7	8.9	8.1	1.3	1.3	1.2	0.9
LB.1	73	12,675	16,166	6.9	7.3	6.3	6.3	0.9	0.9	0.6	0.6
JN.1.18	89	6318	7962	2.3	2	1.7	1.2	1.1	1	1.2	0.9

**Table 3:** Regional prevalence (%) of SARS-CoV-2 variants XEC and KP.3.1.1 across WHO regions during epidemiological weeks 34 and 37 of 2024. The table presents aggregated prevalence data by WHO region for variants under monitoring (VuM) XEC and KP.3.1.1, as reported in the GISAID and WHO variant surveillance summaries. The prevalence percentages reflect the proportion of submitted sequences in each region during the specified weeks. Data from Southeast Asia, Africa, and the Eastern Mediterranean were not reported, or only isolated sequences were included. Data were accessed in compliance with GISAID’s Terms of Use (https://www.gisaid.org/, accessed on 30 June 2025).

VUMs	Region	Epiweek 34 *	Epiweek 37 *
XEC	Europe	5.3	12.0
	The Western Pacific	0.2	2.0
	The Americas	0.9%	2.8%
	Eastern Mediterranean region, Africa, Southeast Asia	Not reported	Not reported
KP.3.1.1	Europe	48.2	50.4
	The Western Pacific	13.5	24.2
	The Americas	34.1	49.2
	Southeast Asia	A single reported sequence	A single reported sequence

**Table 4:** Country-level distribution of SARS-CoV-2 variant XEC based on publicly submitted genomic sequences. This table summarizes the total number of sequenced SARS-CoV-2 cases, and the corresponding number of sequences identified as the XEC variant across 28 countries, as reported in GISAID through Epiweek 47 of 2024. The highest numbers of XEC sequences have been reported in the United States, Canada, and the UK, with XEC also detected at lower levels in several European and Asia–Pacific countries. The data reflect raw sequence counts and does not indicate prevalence relative to total COVID-19 case counts.

Country	No. of All Sequenced SARS-CoV-2 Cases	No. of XEC Cases
Netherlands	10,440	69
Denmark	11,425	92
Slovenia	1483	19
Germany	6891	99
Czech Republic	542	8
Italy	7065	35
France	21,277	110
Sweden	12,910	72
Austria	2238	13
Ireland	5475	36
UK	43,625	122
Portugal	1749	5
Luxembourg	1483	5
Poland	2377	9
Croatia	970	4
Israel	4370	5
Belgium	3367	4
Australia	16,260	22
Finland	2699	4
Canada	64,554	140
Spain	26,388	61
Norway	768	2
Hong Kong	972	1
USA	186,795	168
Taiwan	1399	1
South Korea	20,753	4
Japan	28,004	3
Brazil	10,547	1
China	17,640	1

**Table 5:** Age distribution of SARS-CoV-2 variants XEC and KP.3.1.1, other Omicron lineages, and recombinant variants. This table summarizes the proportional detection of selected SARS-CoV-2 variants across age groups, based on aggregated case-level data reported in late 2024. Values represent the percentage of variant-specific cases within each age category, spanning from infancy (0–4 years) to elderly populations (80+ years). XEC and KP.3.1.1 show broadly consistent detection patterns across age groups, whereas other Omicron lineages and recombinant variants were more commonly identified in younger individuals and were less prevalent overall.

Variants	Ages 0–4	Ages 5–11	Ages 12–19	Ages 20–39	Ages 40–59	Ages 60–79	Ages 80 and Over	Total
XEC	32.3%	25.0%	26.7%	27.8%	32.7%	32.4%	31.9%	31.9%
KP.3.1.1	30.1%	25.0%	13.3%	38.3%	30.7%	33.1%	31.9%	32.3%
Other Omicron	18.3%	25.0%	20.0%	13.6%	16.5%	11.0%	11.2%	11.9%
Other recombinant	1.1%	0.0%	0.0%	1.2%	1.2%	1.1%	1.0%	1.0%

**Table 6:** Age-specific distribution of SARS-CoV-2 variants under monitoring (VuM) reported in late 2024. This table presents the relative prevalence (%) of infections caused by selected SARS-CoV-2 variants XEC and KP.3.1.1, other Omicron sublineages, and other recombinant lineages stratified by age group. The proportions represent the percentage of variant-associated infections within each specified age category, from 0 to 4 years to 80 years and older. XEC and KP.3.1.1 exhibited broadly similar age profiles, while other Omicron and recombinant lineages were less frequently detected across all age groups.

Variants	Age 0–4	Age 5–11	Age 12–19	Age 20–39	Age 40–59	Age 60–79	Age 80 and over	Total
XEC	32.3%	25.0%	26.7%	27.8%	32.7%	32.4%	31.9%	31.9%
KP.3.1.1	30.1%	25.0%	13.3%	38.3%	30.7%	33.1%	31.9%	32.3%
Other Omicron	18.3%	25.0%	20.0%	13.6%	16.5%	11.0%	11.2%	11.9%
Other recombinant	1.1%	0.0%	0.0%	1.2%	1.2%	1.1%	1.0%	1.0%

## Data Availability

No new data was generated from this work.
